# 2463. Bacterial Infections in Intensive Care Unit patients in a large general hospital, Kazakhstan, 2022

**DOI:** 10.1093/ofid/ofad500.2081

**Published:** 2023-11-27

**Authors:** Ademi Yergaliyeva, Saya Gazezova, Gulzhan Ayapova, Elvira Ibragimova, Dilyara Nabirova, Roberta Horth

**Affiliations:** Central Asia Field Epidemiology Training Program, Almaty, Almaty, Kazakhstan; Central Asia Field Epidemiology Training Program, Almaty, Almaty, Kazakhstan; Central Asia Field Epidemiology Training Program, Almaty, Almaty, Kazakhstan; Central Asia Field Epidemiology Training Program, Almaty, Almaty, Kazakhstan; CDC Central Asia office, Almaty, Almaty, Kazakhstan; US Centers for Disease Control and Prevention, Dulles, Virginia

## Abstract

**Background:**

Between January and April 2022, an audit of surveillance data revealed five cases of bloodstream *Acinetobacter baumannii* infection in intensive care unit (ICU) patients in a large general hospital. Bacterial infections such as *Acinetobacter baumannii* contribute to significant mortality and complications of treatment in ICU patients. We aimed to describe bacterial pathogens and their resistance profile among ICU patients to inform infection prevention and control programs.

**Methods:**

We conducted a cross-sectional study of patients admitted to the ICU of a large general hospital in Kazakhstan from January to April 2022. The study included all patients ≥ 18 years old diagnosed with a bacterial infection as detected in blood, urine or sputum samples. Demographic, clinical, and laboratory data were abstracted from medical records.

**Results:**

Among 65 patients identified, 54% were male, mean age 64 years (range 20-93). Majority (35%) were admitted directly to the ICU, 15% had been transferred to the ICU from the infectious surgery department, 14% from the stroke department, and 5% from the gynecology department. Sixty-eight percent (44/65) of patients had surgery prior to transfer to the ICU. The most frequent pathogen in the study patients was *Acinetobacter baumannii* 63% (n=41). Of which, 15% was detected in blood, 41% in urine, and 44% in sputum. The hospital only tests for drug-resistance for the prescribed antibiotic, and 63% (26/41) of strains tested were resistant to their prescribed medicines. *Klebsiella pneumoniae* was detected in 22% (n=15) patients. Of which, 60% was detected in sputum, 20% in urine and 20% in blood. Also, 60% were drug-resistant. *Staphylococcus aureus* was detected in 11% of patients. Of which, 57% was drug-resistant in sputum. Overall 60% (39/64) of pathogens tested had resistance for the antibiotic prescribed, but there was no evidence that prescribed antibiotics changed based on resistance results.

Antimicrobial resitance and antibiotics prescribed to patients in an ICU of a large general hospital (n=65), Kazakhstan, 2022

**Figure:**
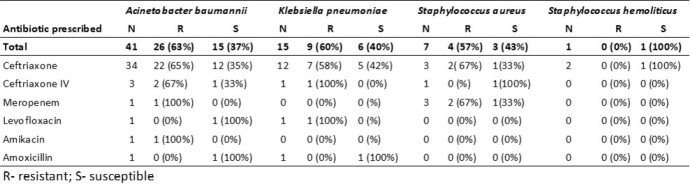

Map of bacterial infection in ICU patients admitted from different wards in a large general hospital, Kazakhstan, 2022

**Figure:**
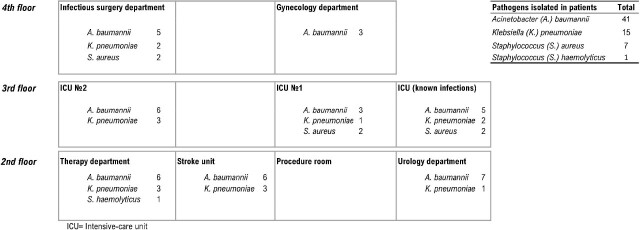

**Conclusion:**

The identification of infectious pathogens with demonstrated drug resistance reinforces the need for improved basic infection control practices and the need for improved antimicrobial stewardship. Increased surveillance and reporting of hospital-acquired infections is needed.

**Disclosures:**

**All Authors**: No reported disclosures

